# Supplementary material for: Mechanisms governing the pioneering and redistribution capabilities of the non-classical pioneer PU.1
Source: Nat Commun. 2020 Jan 21;11:402. doi: 10.1038/s41467-019-13960-2 (PMC6972792; doi:10.1038/s41467-019-13960-2)
Supplement: Supplementary file 7 — Source data [file 41467_2019_13960_MOESM7_ESM.zip › Source_Data/FigureS2/FigureS2B_MotifScanOutput/KmeansCluster4/homerResults/motif29.similar.html]

motif29

## Information for motif29

A
T
G
C
A
G
T
C
T
A
G
C
G
T
A
C
T
A
G
C
T
C
G
A
A
C
G
T
C
T
A
G
T
G
C
A
C
G
A
T
  
Reverse Opposite:  

C
G
T
A
A
C
G
T
A
G
T
C
C
G
T
A
A
G
C
T
A
T
C
G
C
A
T
G
A
T
C
G
C
T
A
G
T
A
C
G
  

|  |  |
| --- | --- |
| p-value: | 1e-33 |
| log p-value: | -7.774e+01 |
| Information Content per bp: | 1.700 |
| Number of Target Sequences with motif | 183.0 |
| Percentage of Target Sequences with motif | 4.05% |
| Number of Background Sequences with motif | 536.2 |
| Percentage of Background Sequences with motif | 1.28% |
| Average Position of motif in Targets | 110.2 +/- 66.1bp |
| Average Position of motif in Background | 98.6 +/- 70.0bp |
| Strand Bias (log2 ratio + to - strand density) | 0.2 |
| Multiplicity (# of sites on avg that occur together) | 1.16 |
| Motif File: | file (matrix) reverse opposite |

### Similar de novo motifs found

|  |  |  |  |  |  |  |  |
| --- | --- | --- | --- | --- | --- | --- | --- |
| Rank | Match Score | Redundant Motif | P-value | log P-value | % of Targets | % of Background | Motif file |
| 1 | 0.807 | C G A T G A T C C T G A G A C T C T A G C T A G A T G C C T A G C T A G | 1e-26 | -61.397422 | 8.73% | 4.70% | motif file (matrix) |
| 2 | 0.799 | T A G C T G C A A T G C T G C A C G A T C A T G G T C A A G C T | 1e-20 | -46.423009 | 4.36% | 1.97% | motif file (matrix) |
